# Groundwater Rise Sustains the World's Largest Alpine Water System Under Global Warming

**DOI:** 10.1002/advs.76957

**Published:** 2026-08-05

**Authors:** Jianqing Du, Zhixiang Niu, Yanfen Wang, Shirui Zhou, Youqing Yang, Xiang Wei, Haishan Niu, Yitao Zhang, Anquan Xia, Yali Liu, Yanbin Hao, Kai Xue, Bingfang Wu, Xinquan Zhao, Tandong Yao, Yiyu Chen

**Affiliations:** ^1^ National Field Observation and Research Station (Beijing Yanshan) for Earth Critical Zone University of Chinese Academy of Sciences Beijing China; ^2^ State Key Laboratory of Earth System Numerical Modeling and Application Chinese Academy of Sciences Beijing China; ^3^ College of Resources and Environment University of Chinese Academy of Sciences Beijing China; ^4^ College of Life Sciences University of Chinese Academy of Sciences Beijing China; ^5^ Development and Research Center China Geological Survey Beijing China; ^6^ School of Grassland Science Beijing Forestry University Beijing China; ^7^ State Key Laboratory of Plateau Ecology and Agriculture Qinghai University Qinghai China; ^8^ State Key Laboratory of Tibetan Plateau Earth System, Environment and Resources (TPESER), Institute of Tibetan Plateau Research Chinese Academy of Sciences Beijing China

**Keywords:** climate change adaptation, groundwater‐vegetation interactions, Qinghai–Xizang plateau, shallow groundwater, vadose zone

## Abstract

Shallow groundwater dynamics on the Qinghai–Xizang Plateau (QXP)−the “Asian water tower” supplying freshwater to billions downstream−remain poorly understood despite their critical role in buffering climate impacts. Integrating more than 8000 in‐situ groundwater records with multi‐source remote sensing data, we present the first high‐resolution assessment of shallow groundwater across the QXP's non‐permafrost plains. From 2000 to 2020, groundwater depth has been decreasing at a rate of 0.02 m year^−^^1^, adding approximately 31.44 Gt (Gigatons) of freshwater storage−directly countering the prevailing narrative of widespread water loss. By combining the maximum capillary rise height, this rise sustains ∼53 500 km^2^ of alpine ecosystems and is closely linked to increasing NDVI in emerging groundwater‐dependent vegetation. These results reveal the possibility of ecosystem regime shift under shrinking groundwater depth, highlighting the importance of groundwater dynamics in understanding alpine ecosystem changes. With an estimated 426.6 Gt of remaining storage capacity in the vadose zone, we identify managed underground reservoirs as a promising but still prospective opportunity for climate adaptation. Our findings reveal that the QXP's shallow aquifers function as a dynamic, growing freshwater reservoir, challenging surface‐water‐centric views of water‐tower vulnerability and suggesting that current studies may underestimate the resilience plateau's water system to ongoing global warming.

## Graphical abstract

Shallow groundwater depth (SGWD) across the non‐permafrost plains of the Qinghai–Xizang Plateau decreased at averagely 0.02 m year^−^^1^, adding 31.44 Gt of freshwater storage from 2000 to 2020 and sustaining ∼53 500 km^2^ of alpine ecosystems. A vadose‐zone capacity of 426.6 Gt reveals these aquifers as promising reservoirs, highlighting groundwater's critical ecohydrological role and climate regulation potential.

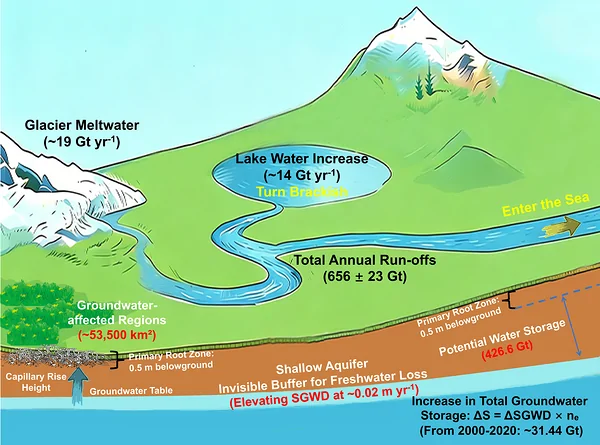

## 1 Introduction

Most of Earth's freshwater is stored in the cryosphere as snow or ice [1]. Under global warming, however, the rapid loss of these solid reserves has intensified over recent decades [2, 3], contributing to an unprecedented freshwater crisis worldwide. A considerable fraction of the melted solid water flows directly into the oceans since most glaciers are located in coastal regions such as the Arctic coasts, Greenland coasts, and Pacific and Atlantic coasts in South America [2, 4, 5]. In contrast, meltwater originating in the headwaters of some densely populated large river basins (e.g., the Qinghai–Xizang Plateau [QXP], known as the “Asian Water Tower”) can still recharge lakes and groundwater [6, 7], raising the prospect of mitigating freshwater losses through strategic water resource management.

The QXP stands at an average altitude of over 4 000 m, sustaining diverse ecosystems [8] and serving as the origin of major river systems across Asia. Over recent decades, the QXP has undergone profound hydrological changes, including glacier retreat [9], snowpack reduction [7], lake expansion [10], runoff increase [11], and permafrost degradation [12]. These changes have led to an accelerating transformation from solid water reserves to liquid water resources, leading to the so‐called “solid–liquid imbalance” [13]. Large amounts of melted solid water resources have either flowed into brackish lakes [14] or flowed out of QXP with a large proportion entering the ocean without being utilized [6, 15]. These phenomena pose serious threats to downstream water security for ∼2 billion people [16].

As an interchange linking solid water and lakes or rivers [17], aquifers−particularly the shallow aquifer in wetlands or other flat areas−serve as critical freshwater reserves [18, 19]. Due to its frequent hydraulic connections with surface water, the shallow aquifer can absorb the melted solid water like a sponge and then release it slowly, thus buffering the rapid freshwater loss. The shallow groundwater changes may also affect vegetation growth and mediate surface eco‐hydrological processes [20]. Therefore, a thorough understanding of the shallow groundwater storage and its changes is essential for the sustainable water management of QXP [21]. Nevertheless, most previous studies on the QXP have generally focused on glacier and permafrost degradation [22, 23] or surface water changes [24, 25], while few studies have focused on shallow groundwater changes. Currently, research on groundwater changes typically utilizes three approaches: interpolation of observed groundwater depth [26], model simulation [27], and analysis of the Gravity Recovery and Climate Experiment (GRACE) data [28, 29]. In regions lacking observed groundwater depth such as the QXP, GRACE is widely used to estimate groundwater changes [30, 31]. Although GRACE could provide invaluable insights into regional groundwater changes, it has several disadvantages in delivering accurate groundwater assessments on the QXP. First, non‐hydrological signal contamination such as high erosion rates can substantially overestimate the regional water storage loss [32]. Second, GRACE has coarse spatial resolution (∼300–400 km), which may blur signals across the QXP's heterogeneous basins and restrict the effective management of local‐scale groundwater resources. Furthermore, it faces great challenges in decomposing vertical signals such as snow/ice, surface water, soil water, and groundwater, particularly separating shallow groundwater from those deep‐buried. Therefore, groundwater changes based on GRACE may exhibit contrasting trends compared with studies based on other methods. For instance, Gao et al. [30] constructed a 0.1° resolution groundwater storage anomaly (GWSA) dataset on the QXP based on GRACE downscaling data and revealed a declining groundwater storage from 2002 to 2020. However, combining multi‐source datasets and using water balance analysis, Zou et al. [33] suggested that groundwater storage on the QXP increased at 5.59 ± 1.44 Gt year^−1^ from 2003 to 2016. These contrasting results underscore the urgent needs of assessing groundwater changes based on monitoring data of regional groundwater depth.

Due to the limited number of monitoring wells, traditional spatial interpolation methods are difficult to apply to investigate groundwater changes on the QXP. Recently, the rapid development of deep learning has shown excellent performance over traditional methods in identifying complex factors and non‐linear processes [34], particularly suitable for estimating groundwater changes which are directly and indirectly influenced by various factors [35]. For example, many studies modeled the change of groundwater depth in European countries using either the Long Short‐Term Memory (LSTM) deep neural network [36, 37] or the convolutional neural network (CNN) [38, 39]. The LSTM models capture long‐term temporal dependencies and nonlinear dynamics in groundwater depth but struggle to model spatial heterogeneity among monitoring wells. Conversely, CNN models can efficiently extract local spatial features and reduce the risk of gradient instability under relatively limited training data. Overall, in the case of scattered spatial distribution of monitoring wells and the lack of long‐term observation data, CNN models generally outperform LSTM models in terms of accuracy and computational speed, while exhibiting higher flexibility and modeling stability compared to nonlinear autoregressive models [40]. These studies have provided valuable experiences for analyzing the shallow groundwater dynamics on the QXP.

Based on the monitoring wells from China's National Groundwater Monitoring Project [41], this study adopts the CNN model with 11 variables involving climate, vegetation, soil, and anthropogenic activity factors to estimate the recent two decades’ shallow groundwater depth (SGWD) of QXP's plain area−the critical but overlooked freshwater reserves. Notably, our study area does not include the permafrost regions. These regions generally have inactive hydrological processes, as permafrost can isolate groundwater from surface interaction and affect groundwater connectivity to some extent [42]. Overall, this study presents a high‐resolution shallow groundwater dynamic on QXP's non‐permafrost plains for the first time. Our findings underscore the pivotal role of shallow aquifers in mitigating freshwater loss across the QXP. Moreover, we uncover a close but complex relationship between SGWD and regional ecosystems via the SHapley Additive exPlanations (SHAP) values, highlighting the importance of exploring groundwater‐induced tipping points and their potential impacts on vegetation succession and climate change in these critical but fragile alpine ecosystems.

## 2 Results

### 2.1 Change of Shallow Groundwater Depth

The grid‐scale and watershed‐scale SGWD changing trends of the annual, wet season, and dry season from 2000 to 2020 were analyzed using the Theil‐Sen median method and the Mann–Kendall test. Over the 20 years, the SGWD in the study area had become shallower at an average rate of 0.02 m year^−1^ (Figure 1). The shallowing areas accounted for 61.7% of the study area, with 23.1% becoming significantly shallower (Figure 2a; *p<*0.05). The shallowing rate in the Inner Basin was more pronounced (0.04 m year^−1^), particularly to the east of Siling Co (0.13 m year^−1^). The Brahmaputra had a shallowing rate of 0.02 m year^−1^, while its headwater exhibited a higher rate at 0.05 m year^−1^. Tarim and Ganges had a shallowing rate of 0.03 and 0.05 m year^−1^, respectively. On the contrary, the Yangtze River, Mekong, Hexi, and Qaidam had an increasing SGWD at 0.01, 0.01, 0.03, and 0.04 m year^−1^, respectively. Notably, the SGWD changing trends in the Yellow River exhibited a strong spatial heterogeneity. A significant increasing trend was revealed at its headwater and Qinghai Lake at a rate of 0.06 and 0.02 m year^−1^, respectively; while the Zoige exhibited a shallowing trend at 0.02 m year^−1^. The SGWD in other basins had no significant changing trends.

**FIGURE 1:**
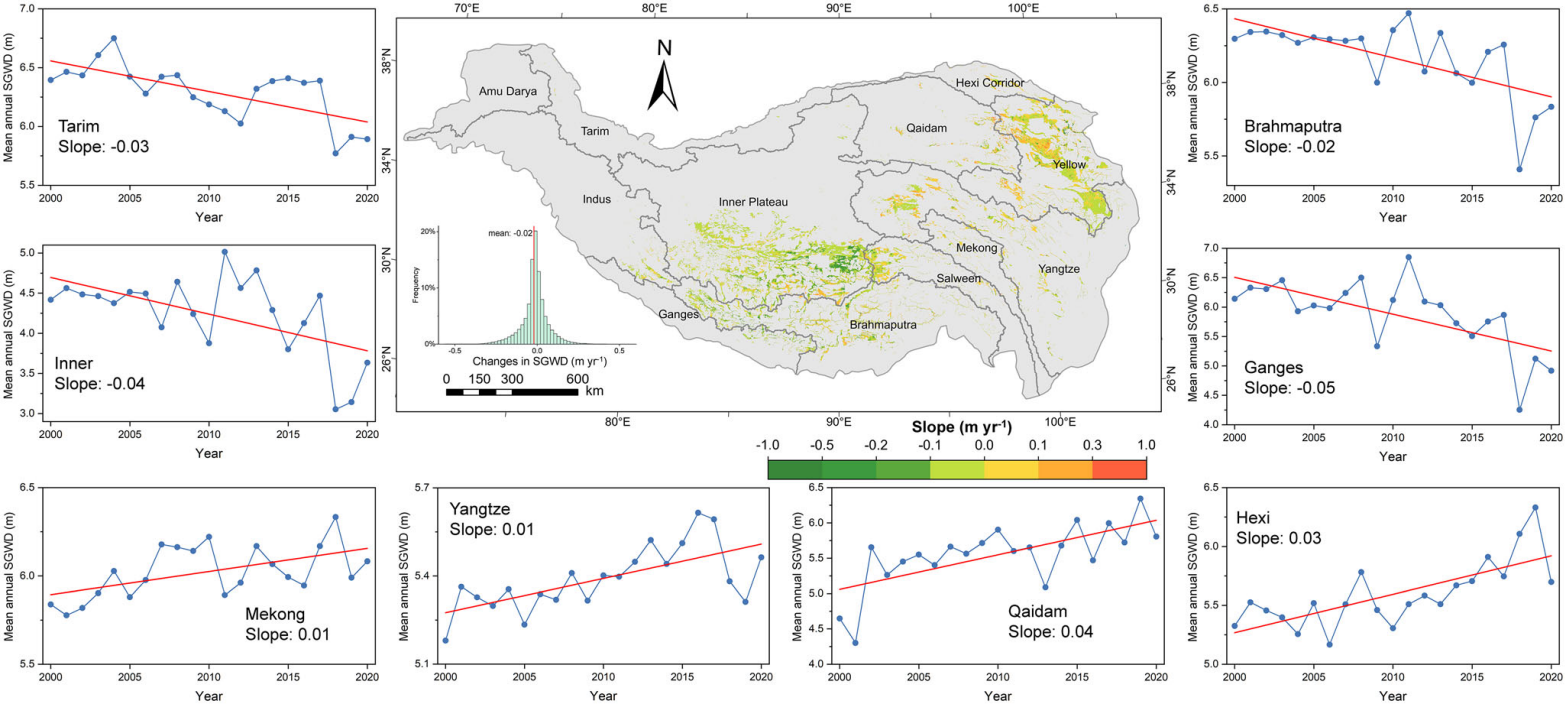
Changes in mean annual shallow groundwater depth (SGWD) from 2000 to 2020. Green areas with negative slope values have decreasing SGWD, and vice versa for red areas. The green bar graph shows the frequency of the mean annual changes. The eight linear fitting graphs illustrate the trends of SGWD in basins with significant changes based on the Mann‐Kendall test.

**FIGURE 2:**
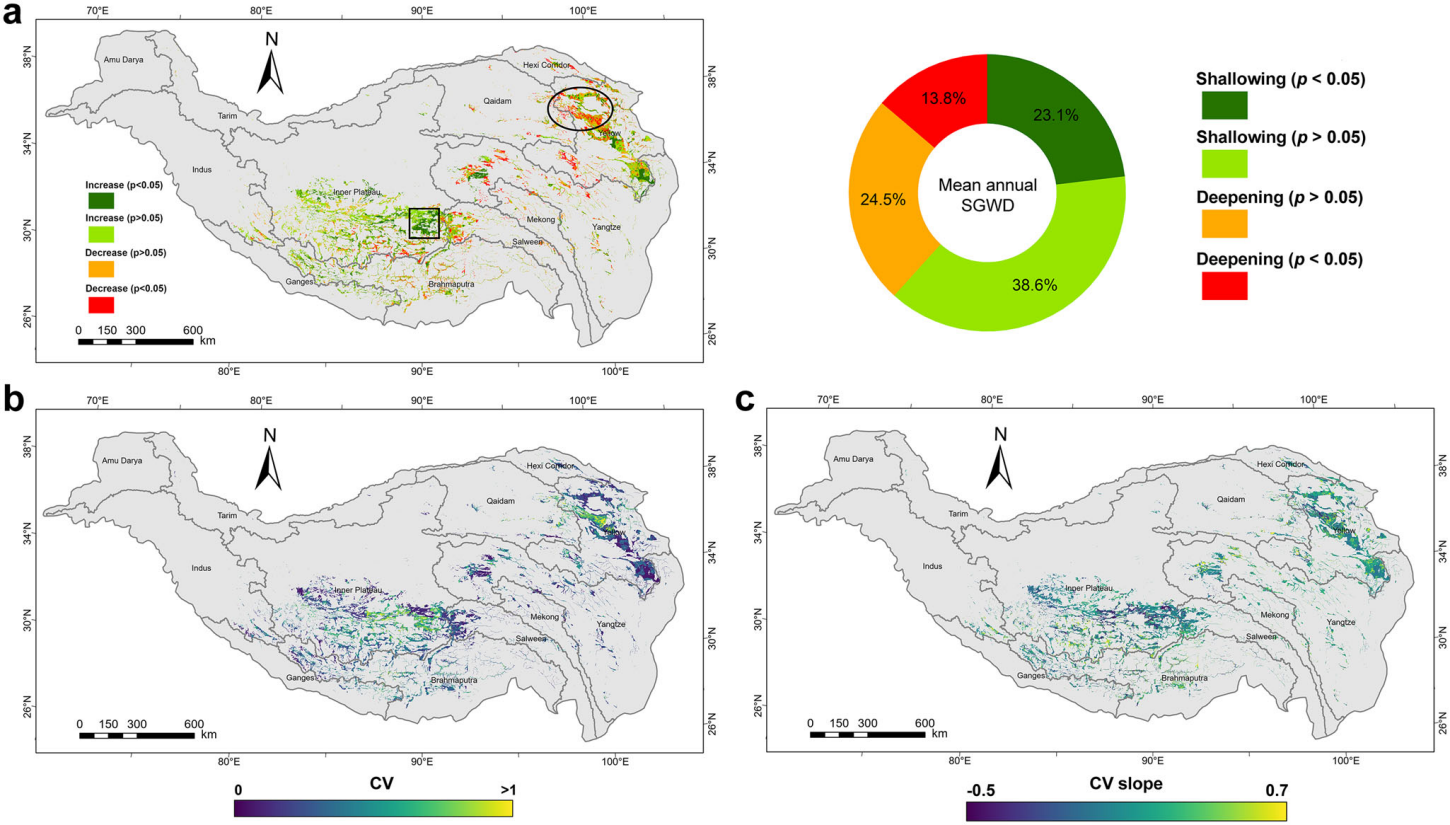
Statistical significance and variability of the changes in shallow groundwater depth (SGWD) from 2000 to 2020. (a) Spatial distribution of the significant and non‐significant changes. (b) The CV of all the mean annual SGWD from 2000 to 2020. c, Changing trends of the intra‐annual CV (calculated by the 12 months’ SGWD within a year). The black rectangle (east Siling Co) and ellipse (south Qinghai Lake) in a refer to the regions with the largest changes.

According to the coefficient of variation (CV) within a year and between years, we explored the intra‐annual and long‐term SGWD fluctuation from 2000 to 2020. Overall, the long‐term SGWD fluctuation was generally low in most regions, but was high in the Inner Basin and the south of Qinghai Lake, where the SGWD exhibited the largest changes (Figure 2b). However, the changing slope of annual CV (calculated by the SGWD of 12 months) was generally positive (Figure 2c), suggesting the intra‐annual SGWD fluctuation was gradually increasing from 2000 to 2020.

Additionally, although both the wet and dry seasons generally exhibited a shallowing SGWD similar to the annual trend (Figures S1 and S2), more areas in the dry season had increasing SGWD compared with the wet season (51.3% vs. 39.0%). This phenomenon is in line with the intensifying intra‐annual SGWD fluctuation as mentioned above.

### 2.2 Change of Shallow Groundwater Storage

Overall, the shallow groundwater storage (SGWS) increased by 31.44 Gt on the QXP from 2000 to 2020. Substantial increases were revealed in the Inner Basin (28.7 Gt, accounting for 91% of the total increase), Brahmaputra (5.5 Gt), Ganges (1.6 Gt), and Yellow River (0.99 Gt) (Figure 3). Notably, a strong spatial heterogeneity was also revealed in the Yellow River Basin. Despite an increase of 3.14 Gt SGWS was found in the Zoige region, large decreases were also revealed around Qinghai Lake. On the contrary, there were also many basins experiencing declining SGWS, namely, the Yangtze River (−2.3 Gt), Qaidam (−1.8 Gt), Hexi Corridor (−0.7 Gt), Mekong (−0.3 Gt), and Salween (−0.2 Gt) (Figure 3). Additionally, the SGWS in the Tarim and Indus basins had changed very little.

**FIGURE 3:**
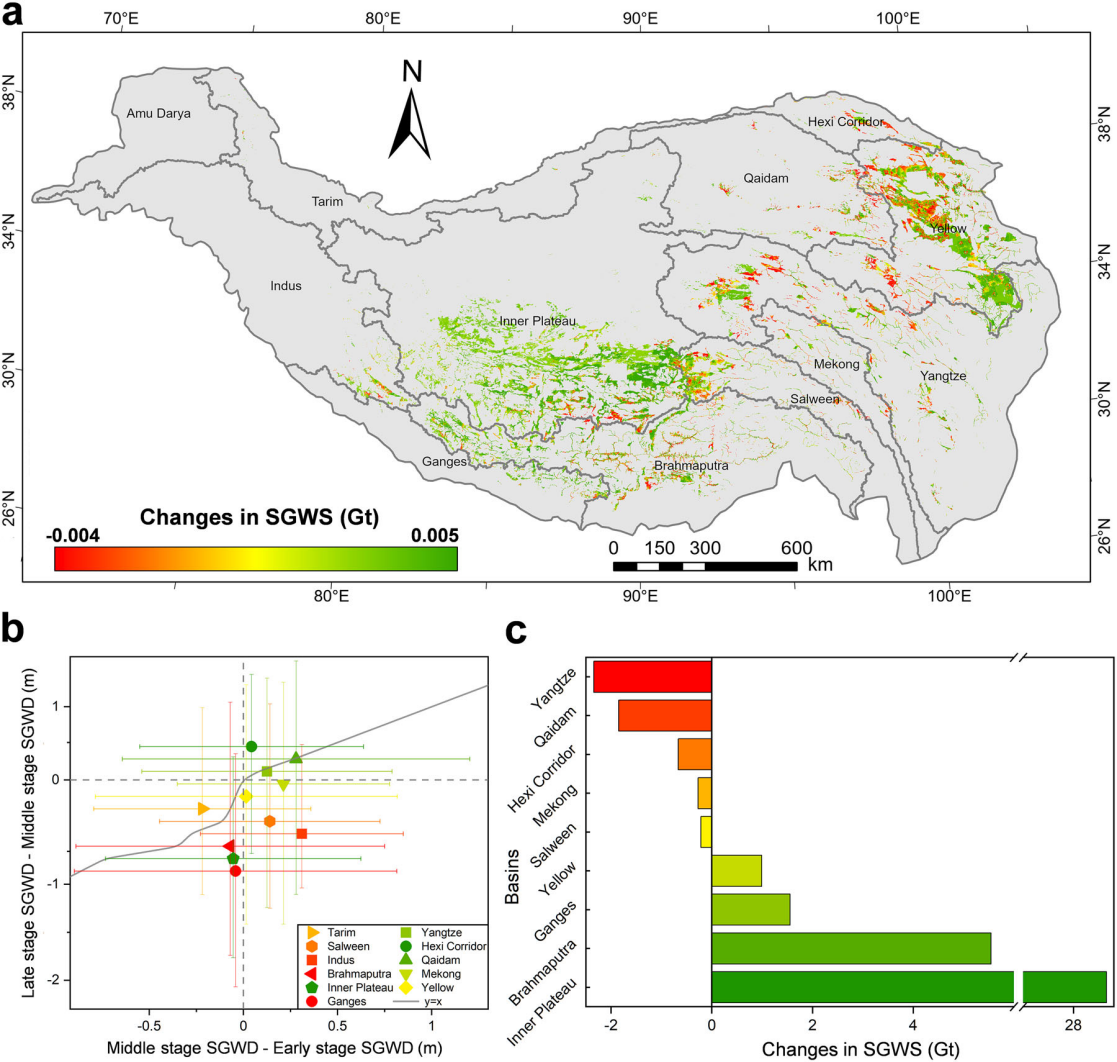
Changes in average shallow groundwater storage (SGWS). (a) Changes from years 2000–2005 to years 2015–2020. (b) Changes of 11 basins’ average SGWD between the early (2000–2005), middle (2006–2014), and late (2015–2020) stages. (c) Changes in SGWS for nine basins (the remaining two basins had almost no changes and are thus not represented). Error bars in b represent one standard deviation.

Moreover, we divided the 20 years into three periods (i.e., early stage [2000–2005], middle stage [2006–2014], and late stage [2015–2020]) to lower the impact of inter‐annual groundwater fluctuations and then compare SGWS changes in different basins (Figure 3b). The increasing trend of the SGWS in the Brahmaputra, Inner, and Ganges basins became faster compared to the middle‐early stage, while it became slower in the Tarim Basin. The declining trends of SGWS in the Hexi Corridor Basin became faster recently, while they changed little in the Qaidam and Yangtze River basins. Notably, the Indus and Salween basins had a declining SGWS from the early to middle stage, but an increased SGWS recently.

### 2.3 The Groundwater‐Affected Regions and Potential Shallow Groundwater Storage

We defined the groundwater‐affected regions (GAs) as those in which the groundwater depth, combined with the maximum capillary rise height, is within 50 cm below the surface (the primary root zone on the QXP) (see Methods for details). The results showed that GAs accounted for 26.3% of the study area in 2020 (∼53,500 km^2^), which is an 18% increase from 2000 (Figure 4). Overall, the GAs were mainly concentrated in the Inner Basin (∼38 400 km^2^) and the Yellow River Basin (∼8,000 km^2^). Notably, GAs in the Inner Basin had expanded by 38% from 2000 to 2020.

**FIGURE 4:**
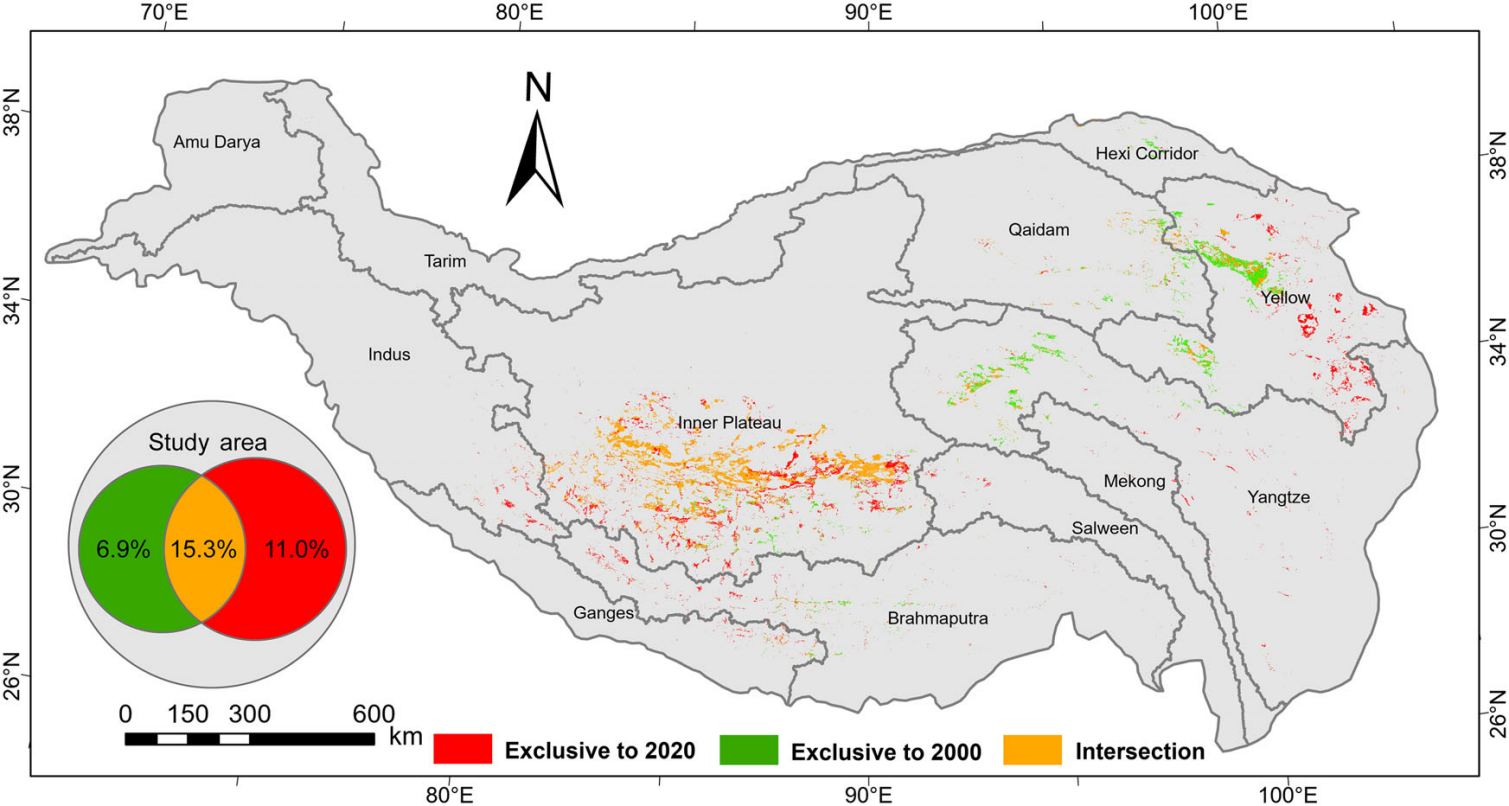
Distribution of the groundwater‐affected regions (GAs). The green and red areas represent the exclusive GAs in 2000 and 2020, respectively, while the orange areas indicate the intersecting regions in both years. The Venn diagram illustrates the area proportion of each section.

Moreover, we estimated the potential groundwater storage from the water table depth to 50 cm below the surface (see Methods for reasons). The potential storage of shallow groundwater on the QXP exhibits pronounced spatial heterogeneity (Figure 5 and Table S1). The total potential groundwater storage in the study area was estimated at 426.59 Gt in 2020. Regions of high potential are mainly concentrated in the northeastern and southern QXP, forming relatively continuous zones along major river basins, whereas low‐potential regions are predominantly distributed across the central interior regions. The Yellow River Basin contains the largest potential storage, reaching 139.80 Gt, followed by the Inner Basin (103.96 Gt) and the Brahmaputra Basin (62.48 Gt). The remaining potential storage is discontinuously distributed across the headwater regions of the Yangtze, Mekong, and Salween rivers.

**FIGURE 5:**
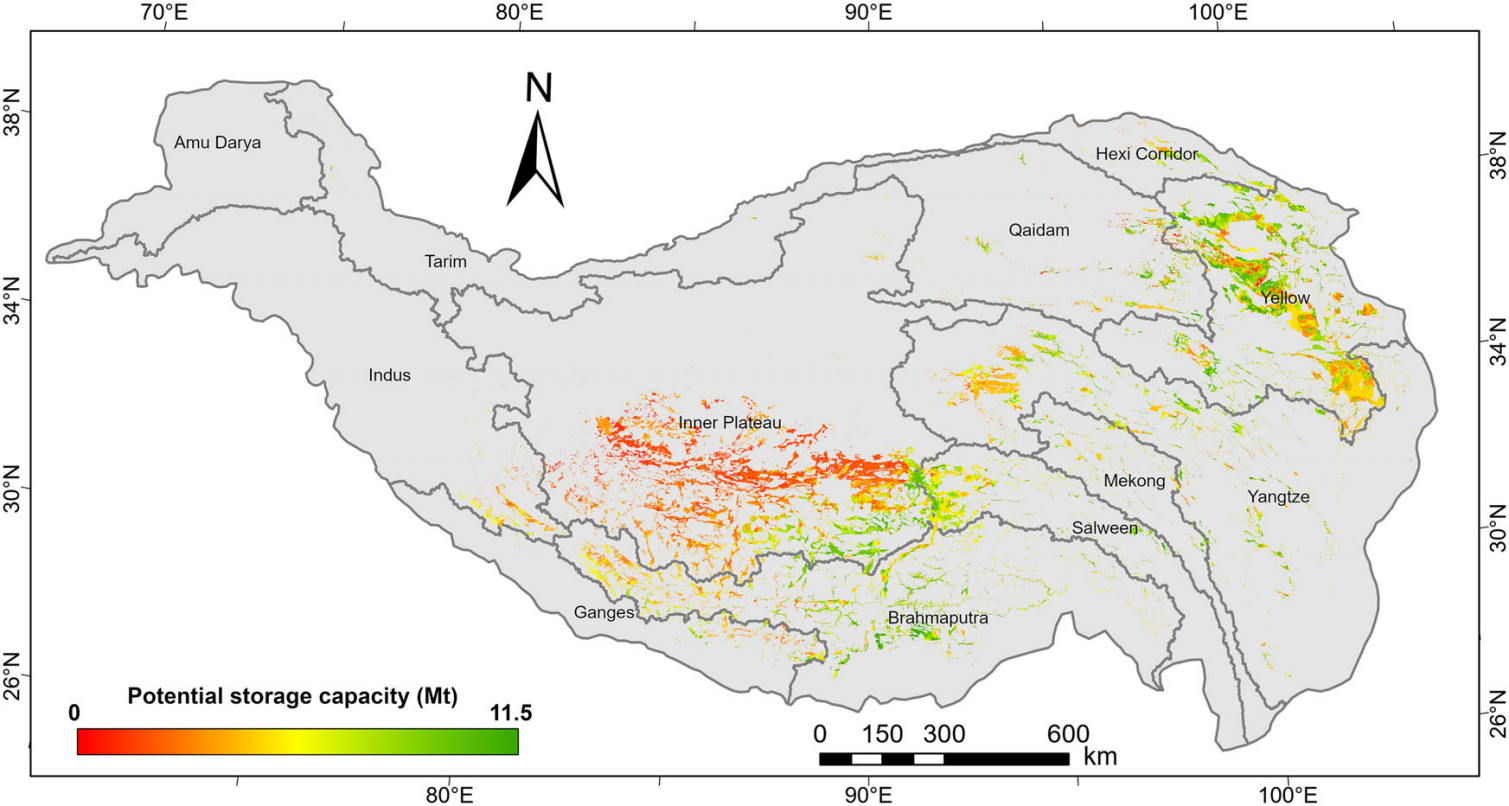
Distribution of the potential storage capacity of the shallow aquifers.

### 2.4 Relative Importance of Selected Variables in the Model

To clarify the relative impact of different potential factors on predicting groundwater variation, we provided interpretability to the model by calculating the SHAP values of each selected variable (see Methods for details; Figure 6a). The feature importance analysis showed that anthropogenic activities were identified as the most important predictive factor of shallow groundwater depth (contributed 48.4% in total), while vegetation and meteorological factors contributed 24.9% and 22.2%, respectively. The three most important variables contributing to the model were night‐time light (NL), population density (POP), and net primary production (NPP). The following four variables, including potential evapotranspiration (PET), gross primary production (GPP), normalized difference vegetation index (NDVI), and precipitation (Pre), exhibited strong but relatively similar contributions. Evapotranspiration (ET), surface soil moisture (SM), and temperature (Tmp) contributed relatively less to the model (mean |SHAP value| < 1), while land surface temperature (LST) had nearly no contribution.

**FIGURE 6:**
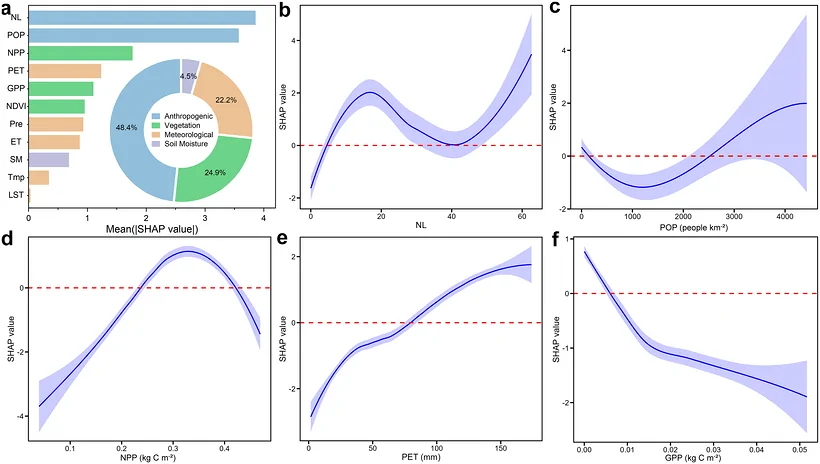
Effects of selected variables on shallow groundwater depth based on the SHapley Additive exPlanations (SHAP). (a) Relative feature importance of each variable. (b–f) SHAP dependency plot of night‐time light (NL), population density (POP), net primary production (NPP), gross primary production (GPP), and potential evapotranspiration (PET), respectively. Positive (negative) SHAP values indicated a positive (negative) contribution of the variable to the shallow groundwater depth. The smooth regression fitting results with a 95% confidence interval were presented in (b–f).

How each factor affects shallow groundwater depth was further analyzed (Figure 6b–e and Figure S3). Generally, increasing NL, POP, PET, and ET were associated with greater groundwater depth (indicated by elevated SHAP values), while higher GPP, SM, Tmp, and LST were associated with lower groundwater depth. The relationship between NPP and groundwater depth was complex. The groundwater depth was positively associated with elevated NPP in habitats with relatively low NPP, while the relationship became negative in habitats with relatively high NPP.

Notably, these results do not address causal relationships but instead demonstrate the importance of relevant factors in predicting changes in groundwater depth. Further analyses focused on the newly formed GAs in 2020 revealed a significant 16.8% increase relative to the NDVI levels of 2000 (paired *t*‐test, t = −133.75, *p *< 0.001). Accounting for the co‐variations of temperature and precipitation, the multivariate regression analysis revealed a significant effect of decreasing groundwater depth on the aforementioned NDVI increases (t = 7.59, *p *< 0.001). This suggests that groundwater changes can drive ecosystem changes, calling for in‐depth analysis in the future.

## 3 Discussion

### 3.1 Reliability and Advantages of Our Dataset

Although no regional dataset of the shallow groundwater depth on the QXP has been published previously, there is a global dataset derived from groundwater modelling [27]. This dataset has fewer than five monitoring sites on the QXP. In fact, it only covers 1,143 sites in Asia and 146 sites in China (for comparison, 837 956, 567 946, 83 617, 78 180, and 34 508 sites in Canada, the US, Australia, Western Europe, and South America, respectively). The lack of monitoring data leads to huge uncertainties for the results on the QXP. As such, this dataset has an extremely skewed data distribution on the QXP (σ = 9.70, skewness = 3.24, kurtosis = 14.48). For comparison, the observed data (σ = 5.96, skewness = 0.99, kurtosis = 0.45) and our dataset (σ = 2.97, skewness = 1.12, kurtosis = 1.26) have much better and also similar data distributions. Notably, our dataset generally underestimates the extreme values, which is a common issue of data‐driven models. Additionally, the global dataset generates a much higher proportion (∼40%) of shallow groundwater depth less than 1 m, as it includes surface water features (rivers, lakes, and inundated wetlands) fed by groundwater discharge (depth ≤ 0) that are not sampled by wells. As a result, this dataset has a lower average groundwater depth (6.2 m) compared with the observed data (7.9 m) and our dataset (8.1 m).

Furthermore, our study presents a high‐resolution map for the shallow groundwater change on the QXP (yearly with a 1 km resolution), which cannot be revealed by the aforementioned global dataset [27], as well as traditional analyses based on the water balance or the GRACE data. Notably, groundwater variations derived from GRACE also rely on the water balance approach and generally represent the total groundwater storage, which makes it particularly challenging to isolate shallow groundwater signals. Such high‐resolution changes allow us to discuss the critical role of shallow unconfined aquifers in maintaining regional water balance and affecting ecosystems.

### 3.2 Buffering Role of Shallow Unconfined Aquifers in Freshwater Storage

Our results indicate that the overall average shallow groundwater depth in the non‐permafrost plains of the QXP is becoming shallower from 2000 to 2020. Such a change is inseparable from the rapid warming trend of the QXP [13]. The conversion of large quantities of solid water into liquid water not only expands lakes and increases runoff, but also recharges groundwater. Although some previous research had indicated an increase in groundwater storage [33, 43], others also suggested a declining groundwater storage in contrast [30]. These studies were generally based on GRACE data or water balance analysis without direct monitoring data, thus very likely leading to the existing inconsistent conclusions. Our results show that the shallow unconfined aquifers in the non‐permafrost plains, occupying only 8% of the area of the QXP, have buffered 31.44 Gt of freshwater. This volume accounts for ∼9% of the total glacier mass loss in the Asian Water Tower and ∼10% of the total increase in lake water storage [13, 14]. Notably, however, the water added to these shallow unconfined aquifers is readily usable freshwater, in contrast to the majority of rapidly expanding lakes, which are commonly saline water. The increased groundwater storage is approximately equal to the total storage capacity of the Three Gorges Reservoir and 5.56 times the total water consumption of Qinghai and Xizang in 2020, demonstrating a huge potential for freshwater supply.

Overall, there was still a 426.6 Gt water storage capacity (theoretical storage capacity of the unsaturated zone) in these shallow unconfined aquifers, which may play a critical role in alleviating QXP's rapid freshwater loss under appropriate engineering measures. Regions with low potential, such as the Inner Basin, generally had shallow groundwater depth, which limits their theoretical water storage capacity per unit area. Notably, the actual utilization of this potential is constrained by multiple factors, including groundwater recharge rate, local topography, aquifer structure, engineering and economic feasibility, and potential ecological risks, which calls for more systematic and comprehensive hydrological, engineering, ecological, and economic assessments.

Furthermore, the high spatial resolution of our results allows for analyzing the spatial heterogeneity of groundwater changes. The Inner Basin and Brahmaputra Basin in Xizang had the fastest increasing shallow groundwater storage in the study period, roughly accounting for 21% and 4% of Brahmaputra's mean annual runoff. These two basins are also the regions experiencing the strongest glacier melting [44], underscoring the importance of shallow unconfined aquifers in buffering the QXP's freshwater loss. For the Inner Basin, although previous studies suggested increasing glacier melting and surface water storage in this region [16, 24], it had been reported that the groundwater storage was declining due to the deep groundwater leakage caused by the Qiangtang Rift Valley [43, 45]. However, unlike these studies based on GRACE, our study focused on changes in shallow groundwater and highlighted that its storage in this region is on a rapid rise, leading to ∼38 000 km^2^ of arid ecosystems potentially affected by shallow groundwater in the future. As for the Brahmaputra Basin, a significant imbalance among precipitation, meltwater, evapotranspiration, and runoff was revealed by previous research [46], which may be attributed to the elevated shallow groundwater storage. The regions that had the largest decline in shallow groundwater storage were the headwaters of the Yellow River (Maduo County) and the south of Qinghai Lake (Gonghe County) in Qinghai. These two counties had a rapidly growing gross domestic product (GDP) from 2004 to 2020 (∼1400% increase for Maduo County and ∼800% increase for Gonghe County), which is very likely the driver of rapidly decreasing shallow groundwater storage. Nevertheless, such a decline was overridden by the increasing shallow groundwater storage of the Zoige region, leading to an overall increase of 0.99 Gt groundwater in the Yellow River Basin (accounting for ∼2% of the Yellow River's mean annual runoff and ∼40% of Qinghai Province's total water consumption in 2020).

### 3.3 Reasons and Ecological Implications of Shallow Groundwater Changes

Our study demonstrates that shallow groundwater change is not only affected by anthropogenic activities and climate, but also strongly associated with ecosystem changes. It is not surprising that anthropogenic activities are the most significant factors driving shallow groundwater changes. Apart from population, night‐time light intensity primarily reflects the intensity of human activities, the degree of urbanization, and the level of socioeconomic development, and can therefore serve as an indirect proxy for anthropogenic pressure on groundwater systems [47]. Regions with higher night‐time light intensity are generally characterized by greater population density, more intensive economic activities, and higher agricultural and industrial water demand, which collectively contribute to increasing groundwater depth. However, when nighttime light intensity is low, groundwater changes are not sensitive to it, and this finding is also consistent with the response of groundwater changes to population density. For climate factors, evapotranspiration is a vital discharge pathway for shallow groundwater, and thus increases groundwater depth. The elevated temperature in the alpine zone may cause the melting of snow, glaciers, and permafrost, which increases the recharge of shallow groundwater. However, the influence of precipitation on groundwater changes on the QXP is weak because of the relatively thick vadose zone, as part of the precipitation may first replenish the water‐holding capacity of the unsaturated vadose zone. Meanwhile, the frequently occurring heavy rainfall events on the QXP [48] can cause infiltration‐excess runoff and prevent the rainwater from entering the shallow aquifers. In general, the groundwater depth declined across ecosystems characterized by different precipitation regimes and vegetation types. This trend had even been observed in arid regions experiencing reduced precipitation (i.e., Brahmaputra Basin), likely driven by enhanced glacial and permafrost meltwater recharge under climate warming. These findings underscore the complex impacts of climate change on groundwater systems [13, 22].

Notably, our results uncover a strong interaction between ecosystem changes and groundwater depth, which is seldom reported in grassland ecosystems at the regional scale. Generally, plant growth is positively associated with shallowing groundwater depth. This conclusion is further supported by the analysis of groundwater‐affected regions based on both groundwater depth and maximum capillary rise height. It is revealed that groundwater could reach the root layer in 26.3% of the study area, which is an 18% increase since 2000 and has significantly affected regional plant growth. These areas are mainly distributed in the Inner Basin and the Yellow River Basin, which is consistent with the recent mapping of global groundwater‐dependent ecosystems [49, 50]. These findings underscore the potential of ecosystem regime shifts in response to shallowing groundwater depth, particularly in the arid Inner Basin. In fact, areas with the same dominant plant species but differing groundwater depths have already undergone succession into distinct ecosystems (Figure S4; *Carex moorcroftii* grassland and wetland under deep and shallow groundwater depth, respectively). Such changes may enlarge the regional carbon sink while elevating ecosystems’ water consumption, thus mediating regional water and carbon cycling and further affecting global climate change [51]. On the contrary, beyond conventionally acknowledged factors such as overgrazing and drought, the rapid depletion of groundwater resources in the headwaters of the Yellow River and the south of Qinghai Lake may also trigger regional ecosystem degradation. Nevertheless, current research mainly focuses on the impacts of increasing groundwater depth [28, 52], while neglecting the potential effects of groundwater depth changes on ecosystems and even global climate change.

## 4 Conclusions

This study presents the first high‐resolution assessment of shallow groundwater dynamics across the QXP, revealing its critical but previously unquantified role in buffering the ongoing imbalance between solid and liquid water in the Asian Water Tower. Notably, the uneven spatial distribution of monitoring wells may still introduce uncertainties into our results, despite our efforts to incorporate multi‐source, spatiotemporally continuous environmental variables into the SGWD reconstruction framework to improve regional representativeness [53]. Therefore, it is essential to establish a long‐term, spatially representative groundwater monitoring network to strengthen future large‐scale groundwater assessments.

Beyond documenting the shallowing of groundwater depth and a substantial increase in freshwater storage, this study further identifies a vast and usable capacity within the vadose zone, positioning shallow aquifers as a strategic natural reservoir for climate adaptation. Importantly, the observed linkages between groundwater and alpine ecosystems underscore the potential regime shift under global warming, yet their mechanisms and influences remain inadequately investigated. Future research should integrate data‐driven results with eco‐hydrological models to reveal the causal pathways and critical points of groundwater‐ecosystem interactions, thereby laying a solid foundation for constructing potential climate feedback models [54]. Collectively, our findings highlight the urgent need to integrate adaptive groundwater management strategies into climate actions, which may simultaneously support water sustainability, ecological conservation, and carbon sequestration.

## 5 Methods

### 5.1 Study Area

Plain regions generally have widely distributed shallow aquifers [18]. These aquifers have close hydraulic connections with surface waters, which may act as critical “water exchange hubs” to store meltwater and release it gradually to buffer the freshwater loss on the QXP. Simultaneously, shallow groundwater dynamics could also directly affect vegetation growth and surface eco‐hydrological processes [20]. Therefore, we chose QXP's plain regions (surface relief less than 30 m) [55] as our study area. We excluded the permafrost regions because they generally have inactive hydrological processes [42] and very few groundwater monitoring wells due to the sparse population. As such, the study area was finalized as a 203 500 km^2^ non‐permafrost plain region (Figure S5).

### 5.2 Data Source

The monthly monitoring data for the groundwater depth were based on the ∼300 wells of China's National Groundwater Monitoring Project from January 2018 to August 2021 [41]. All wells were located in the shallow unconfined aquifers, with a SGWD of less than 30 m in almost all wells.

According to recent studies [36, 38, 40], the factors that exhibited a strong relationship with groundwater changes can be classified into four types: climate, vegetation, soil, and anthropogenic activity. For climate factors, land surface temperature (LST), temperature (Tmp), precipitation (Pre), evapotranspiration (ET), and potential evapotranspiration (PET) were chosen. For vegetation factors, we used the normalized difference vegetation index (NDVI), gross primary production (GPP), and net primary production (NPP). Anthropogenic effects on groundwater are more pronounced in densely populated areas. Thus, population density (POP) and night‐time light (NL) were selected. Surface soil moisture (SM) may also reflect groundwater change and was chosen as another dimension. More information regarding the source, resolution, and processing method was provided in the supplementary methods and Table S2.

### 5.3 Data Processing

Monthly data were used in modeling. For the data recorded annually (e.g., NL and POP), all months within a year were set to the same value. Few short‐term missing values were filled in by linear interpolation, while long‐term missing values (more than three months) were excluded from modeling. Finally, all data (including the SGWD data and the 11 variables) were uniformly log‐transformed before modeling.

### 5.4 Model Training and Validation

The convolutional neural network (CNN) was chosen for this study due to its better performance in groundwater modeling [40]. The model consisted of an input layer, a convolutional layer, a pooling layer, and a fully connected layer. The input layer for this study was the 44 months of SGWD data from the ∼300 monitoring wells and the 11 selected variables. For each groundwater monitoring well, the model input data of the 11 variables were averaged over the well's surrounding 1 km × 1 km grid.

A one‐dimensional CNN approach was used to train the model, while a total of five convolutional layers with a convolutional kernel of three were defined. Each convolution operation was followed by a maximum pooling layer with a pooling window of size two. To diminish computational complexity and prevent overfitting, the data dimensionality was lowered by reducing the length of each feature map. In addition, a Dropout layer with a probability of 0.5 was added after the last convolutional layer to randomly discard half of the neurons during training to prevent model overfitting. The relative importance of each variable in predicting SGWD was calculated by the SHapley Additive exPlanations (SHAP) values. The above processes used the packages PyTorch, GDAL, and SHAP in Python.

For validation, all data were randomly divided into two parts, where 80% was used as the training set (7024 samples) and the remaining 20% was used as the validation set (1756 samples). We calculated the relative error (RE) between observed values (R) and predicted values (P) as the measure of model performance: $RE=|\frac{R-P}{R}|$. In addition, the coefficient of determination (R^2^) was also calculated. After continuous improvement of the model, the final R^2^ was 0.64 (Figure S6a; based on the validation set). We did not aim for a higher R^2^, which would have overfitted the model and made it less generalizable in estimating regional SGWD. In spatially heterogeneous groundwater systems, extremely high R^2^ values may reflect the model learning site‐specific noise rather than capturing transferable hydrological relationships. Such overfitting can reduce inter‐site variability in predictions, leading to an artificially constrained value range. Overall, the RE of the ∼70% validation set is less than 0.4, and half is less than 0.2, suggesting a good performance of the model compared with existing studies [56, 57] (Figure S6b). A flow chart of the modeling processes was provided in Figure S7.

### 5.5 Analysis of the Changes in Groundwater Depth and Storage

The SGWD changing trends of the annual, wet season, and dry season from 2000 to 2020 were analyzed using the Theil‐Sen median method and the Mann‐Kendall test (see Movie S1 for yearly spatial distribution). Because shallow groundwater dynamics are strongly constrained by topography, recharge‐discharge processes, and river networks, we adopted the watershed as a spatially meaningful unit for characterizing spatial heterogeneity. We divided the QXP into 12 basins, namely, Inner Basin, Ganges, Brahmaputra, Indus, Amu Darya, Salween, Mekong, Yellow River, Yangtze River, Tarim, Qaidam, and Hexi Corridor. The areas with limited results, including the northern Inner Basin, Indus, and Amu Darya, were primarily characterized by extensive permafrost distribution or limited plain terrain. Additionally, parts of the Qaidam and Tarim were also excluded due to the lack of model input data (i.e., evapotranspiration [ET]). Moreover, we calculated the interannual and intra‐annual coefficient of variation (CV) to depict the long‐term and annual SGWD fluctuation from 2000 to 2020. All results were displayed using the Albers projection.

The change of shallow groundwater storage (SGWS) of a grid was calculated by multiplying the ∆SGWD by the soil porosity. We used the second version of the China dataset of soil properties for land surface modeling [58] to obtain the grid‐level soil porosity through weighted averaging of all deep layers (layer depth > 0.3 m). Specifically, soil porosity datasets from multiple layers are integrated via weighted averaging to represent effective vadose‐zone porosity (n_e_). Then, groundwater storage change is calculated as ΔS = ΔSGWD × n_e_, where ΔSGWD represents groundwater depth fluctuation. The ∆SGWD was calculated based on the difference between the average SGWD of the two periods, namely 2000–2005 and 2015–2020.

Potential groundwater storage capacity refers to the theoretical water storage space in the vadose zone, not the currently usable or existing groundwater resources. This capacity refers to the storage space from the average SGWD in 2020 to 0.5 m below the surface (the common active root layer on the QXP) [59]. We excluded the surface layer because its water storage is mainly controlled by rainfall. The porosity was calculated using a weighted average method based on the abovementioned dataset [58]. Because these calculations are based on a single‐year static snapshot and do not incorporate the temporal variability of the water table, there is no associated error interval or range of variation.

### 5.6 Calculation of the Maximum Capillary Rise Height

To explore the potential effects of groundwater change on ecosystems, we further calculated the maximum capillary rise height (MCRH) to identify the regions that can be directly affected by shallow groundwater (where the MCRH reaches the root layer). An efficient method for MCRH estimation at the regional scale is to employ the effective particle size of soils (*D*_10_) combined with an empirical model [60, 61]:

(1) $$h_{c}=-990\ln\left(D_{10}\right)-1540$$

where *h_c_* is the MCRH, and *D*_10_ is the particle size at which 10% of the total soil mass consists of particles smaller than this size. Based on the measured data of ∼300 soil samples from the QXP, a grain size cumulative curve was constructed to estimate *D*_10_:

(2) P=aln(D)+b

where *P* is the cumulative percentage, *D* is the corresponding soil particle size, *a* and *b* are the empirical coefficients of the model. When *P* is 10%, the corresponding *D* is *D*_10_. Overall, the R^2^ of the model is above 0.9, indicating high prediction accuracy (Figure S8). Based on these equations, we utilized the soil texture classification dataset [62] to obtain the spatial distribution of the MCRH on the QXP. Notably, a correction coefficient was adopted to lower the potential overestimation of the MCRH based on Equation (1) [63]. By adding the MCRH to the SGWD, we identified the groundwater‐affected regions (GAs) as those MCRH reaches within 50 cm below the surface.

## Author Contributions

**Jianqing Du**: conceptualization, methodology, formal analysis, data curation, Writing – original draft, review & editing. **Zhixiang Niu**: formal analysis, data curation, visualization, writing – original draft. **Yanfen Wang**: conceptualization, supervision, writing – review & editing. **Shirui Zhou**: Formal analysis. **Xiang Wei, Anquan Xia**: Data Curation, Writing – Review & Editing. **Youqing Yang, Haishan Niu, Yitao Zhang, Yali Liu, Yanbin Hao, Kai Xue, Bingfang Wu, Xinquan Zhao, Tandong Yao, Yiyu Chen**: Writing – Review & Editing.

## Conflicts of Interest

The authors declare no conflicts of interest.

## Supporting information




**Supporting File 1**: advs76957‐sup‐0001‐SuppMat.docx.

**Supporting File 2**: advs76957‐sup‐0002‐SuppMat.docx.

**Supporting File 3**: advs76957‐sup‐0003‐SuppMat.docx.

**Supporting File 4**: advs76957‐sup‐0004‐MovieS1.mp4.

## Data Availability

The data training the model are provided as a supplementary data file.
