# Supplementary material for: Groundwater Rise Sustains the World's Largest Alpine Water System Under Global Warming
Source: Adv Sci (Weinh). 2026 Aug 5;13(60):e76957. doi: 10.1002/advs.76957 (PMC13440378; doi:10.1002/advs.76957)
Supplement: Supplementary file 1 [file ADVS-13-e76957-s001.docx]

**Supplementary Methods for**

**Groundwater rise sustains the world’s largest alpine water system under global warming**

Jianqing Du^†^, Zhixiang Niu^†^, Yanfen Wang^*^, Shirui Zhou, Youqing Yang, Xiang Wei, Haishan Niu, Yitao Zhang, Anquan Xia, Yali Liu, Yanbin Hao, Kai Xue, Bingfang Wu, Xinquan Zhao, Tandong Yao, Yiyu Chen

* Corresponding author: Yanfen Wang (Email address: yfwang@ucas.ac.cn)

† These authors contributed equally to this work.

The monthly precipitation [1] and monthly mean temperature [2] datasets were generated using the Delta spatial downscaling programme based on the CRU’s global 0.5° climate dataset and the WorldClim’s global high-resolution climate dataset. Data from 496 independent meteorological stations were used for validation. The monthly potential evapotranspiration dataset [3] was obtained using the Hargreaves potential evapotranspiration formula based on the 1-km monthly mean, minimum, and maximum temperature datasets for China. Evapotranspiration product [4] is a year-end gap-filled 8-day composite dataset produced at 500 m pixel resolution. The algorithm used for the MOD16 data product collection is based on the logic of the Penman-Monteith equation, which includes inputs of daily meteorological reanalysis data along with MODIS remotely sensed data products such as vegetation property dynamics, albedo, and land cover. The land surface temperature dataset [5] was generated using a satellite thermal infrared remote sensing - reanalysis data integration method based on a novel surface temperature time decomposition model. The method makes full use of the high-frequency component of surface temperature, the low-frequency component, and the spatial correlation of surface temperature provided by satellite thermal infrared remote sensing and reanalysis data. The test results, based on measured data from six stations on the QXP and the Black River Basin, showing that the dataset has a high degree of agreement with the measured LST during both day and night under clear sky conditions. The surface soil moisture dataset [6] prior to 2019 is based on the secondary development of the ESA-CCI active-passive fusion product. A global spatially and temporally continuous surface soil moisture dataset with a resolution of 1 km was generated by fusing ERA5 reanalysis data to obtain a spatially and temporally continuous soil moisture dataset with a coarse resolution (25 km), and then used a machine learning algorithm with the global soil moisture observations provided by the International Soil Moisture Network as the training target. The post-2019 surface soil moisture product [7] is based on a synergy of all-weather surface temperature and reanalysis data. This method analyses the linear relationship between diurnal and nocturnal surface temperature differences and surface soil moisture data under different vegetation densities (sparse, medium, and dense) for the four main land cover types (forest, cropland, grassland, and bare land) in China using ERA5-Land (spatial resolution of 0.1°). The NDVI dataset [8] is based on the Aqua/Terra MODIS satellite sensor MOD13Q1 product and land use data, and is generated through the processes of preliminary reconstruction of similar feature noise pixels from single period imagery, S-G filtering of long time series imagery, retention of high quality pixels, 16-day synthesis of monthly products, and China-wide stitching. The Landsat Gross Primary Production CONUS dataset [9] estimates GPP using Landsat Surface Reflectance for CONUS. The GPP is the amount of carbon captured by plants in an ecosystem and is calculated using the MOD17 algorithm with Landsat Surface Reflectance, gridMET, and the National Land Cover Database. The NPP dataset [10] before 2018 was based on the NPP estimation methodology of the CASA model, after which the NPP dataset [11] was provided by the MOD17A3HGF V6.1 product. Population data [12] was obtained from the WorldPop platform, based on census data for administrative units and multiple sources of data such as land cover, night lighting, topography, distance to roads and rivers, spatialized using a random forest machine learning algorithm. Due to the sparsely populated nature of the QXP and the fact that large areas are predominantly nomadic with no settlements, the unconstrained dataset was used. The Chinese DMSP-OLS-like 1-km night-time light remote sensing dataset [13] was used, which is calibrated using the ‘pseudo-invariant pixel’ method. Considering the consistency of temporal resolution between DMSP-OLS and SNPP-VIIRS data, the missing data in the original monthly SNPP-VIIRS data were corrected before synthesizing the annual SNPP-VIIRS data.

**References**

1.	S. Peng, 1-km monthly precipitation dataset for China (1901-2024). *National Tibetan Plateau / Third Pole Environment Data Center.* (2020). <https://doi.org/10.5281/zenodo.3114194>.

2.	S. Peng, 1-km monthly mean temperature dataset for China (1901-2024). *National Tibetan Plateau / Third Pole Environment Data Center.* (2019). <https://doi.org/10.11888/Meteoro.tpdc.270961>.

3.	S. Peng, 1-km monthly potential evapotranspiration dataset for China (1901-2024). *National Tibetan Plateau / Third Pole Environment Data Center.* (2022). <https://doi.org/10.11866/db.loess.2021.001>.

4.	S. Running, Q. Mu, M. Zhao, et al., MODIS/Terra Net Evapotranspiration Gap-Filled 8-Day L4 Global 500m SIN Grid V061, *NASA Land Processes Distributed Active Archive Center.* (2021). <https://doi.org/10.5067/MODIS/MOD16A2GF.061>.

5.	J. Zhou, X. Zhang, W. Tang, et al., Daily 1-km all-weather land surface temperature dataset for Western China (TRIMS LST-TP; 2000-2024) V2. *National Tibetan Plateau / Third Pole Environment Data Center.* (2019). <https://doi.org/10.11888/Meteoro.tpdc.270953>.

6.	C. Zheng, L. Jia, and T. Zhao, Global daily surface soil moisture dataset at 1-km resolution (2000 - 2020). *National Tibetan Plateau / Third Pole Environment Data Center.* (2022). <https://doi.org/10.11888/RemoteSen.tpdc.272760>.

7.	Y. R. Zhang, G. F. Shang, P. Leng, et al., Estimation of quasi-full spatial coverage soil moisture with fine resolution in China from the combined use of ERA5-Land reanalysis and TRIMS land surface temperature product, *Agric. Water Manage.* 275 (2023): 107990.

8.	J. Gao, Y. Shi, H. Zhang, et al., China regional 250m normalized difference vegetation index data set (2000-2024). *National Tibetan Plateau / Third Pole Environment Data Center.* (2023). <https://doi.org/10.11888/Terre.tpdc.300328>.

9.	N. P. Robinson, B. W. Allred, W. K. Smith, et al., Terrestrial primary production for the conterminous United States derived from Landsat 30 m and MODIS 250 m, *Remote Sens. Ecol. Conserv.* 4 (2018): 264-280.

10.	W. Liu, NPP dataset of remote sensing for ecological assets assessment in Qinghai-Tibet Plateau. *National Tibetan Plateau / Third Pole Environment Data Center.* (2019). <https://doi.org/10.11888/Ecolo.tpdc.270532>.

11.	S. Running and M. Zhao, MODIS/Terra Net Primary Production Gap-Filled Yearly L4 Global 500m SIN Grid V061. *NASA Land Processes Distributed Active Archive Center.* (2021). <https://doi.org/10.5067/MODIS/MOD17A3HGF.061>.

12.	C. T. Lloyd, H. Chamberlain, D. Kerr, et al., Global spatio-temporally harmonised datasets for producing high-resolution gridded population distribution datasets, *Big Earth Data* 3 (2019): 108-139.

13.	Y. Wu, K. Shi, Z. Chen, et al., Developing Improved Time-Series DMSP-OLS-Like Data (1992-2019) in China by Integrating DMSP-OLS and SNPP-VIIRS, *IEEE Trans. Geosci. Remote Sensing* 60 (2022): 1-14.
