# Supplementary material for: Groundwater Rise Sustains the World's Largest Alpine Water System Under Global Warming
Source: Adv Sci (Weinh). 2026 Aug 5;13(60):e76957. doi: 10.1002/advs.76957 (PMC13440378; doi:10.1002/advs.76957)
Supplement: Supplementary file 2 [file ADVS-13-e76957-s002.docx]

**Supplementary Figures and Tables for**

**Groundwater rise sustains the world’s largest alpine water system under global warming**

Jianqing Du^†^, Zhixiang Niu^†^, Yanfen Wang^*^, Shirui Zhou, Youqing Yang, Xiang Wei, Haishan Niu, Yitao Zhang, Anquan Xia, Yali Liu, Yanbin Hao, Kai Xue, Bingfang Wu, Xinquan Zhao, Tandong Yao, Yiyu Chen

* Corresponding author: Yanfen Wang (Email address: yfwang@ucas.ac.cn)

† These authors contributed equally to this work.

Figures S1-8

Tables S1 and S2

**FIGURE S1 | Changes in mean wet season shallow groundwater depth (SGWD)** **from 2000 to 2020. a,** Spatial pattern. **b,** Frequency. **c** and **d,** Distribution and proportion of the significant and non-significant changes.

**FIGURE S2 | Changes of mean dry season shallow groundwater depth (SGWD) from 2000 to 2020. a,** Spatial pattern. **b,** Frequency. **c** and **d,** Distribution and proportion of the significant and non-significant changes.

**FIGURE S3 | The SHAP dependence plot of the other six feature variables.** **a,** Evapotranspiration (ET). **b,** The normalized difference vegetation index (NDVI). **c** Precipitation (Pre). **d,** Surface soil moisture (SM). **e,** Land surface temperature (LST). **f,** Temperature (Tmp). The smooth regression fitting results with a 95% confidence interval were presented.

**FIGURE S4 | *Carex moorcroftii* grassland (a) and wetland (b) under deep and shallow groundwater depth.**

**FIGURE S5 | Distribution of the study area and monitoring wells.**

**FIGURE S6 |** **The CNN model accuracy plots.** **a,** The observed values versus the corresponding predicted values. **b,** Frequency of the relative errors.

**FIGURE S7 | The flowchart of the CNN model.**

**FIGURE S8 |** **The *D*_10_ model accuracy plot.**

**TABLE S1 | Potential water storage of the shallow aquifers in each basin.**

| **Basin** | **Groundwater storage potential (Gt)** |
| --- | --- |
| Yellow | 139.80 |
| Inner Basin | 103.96 |
| Brahmaputra | 62.48 |
| Yangtze | 49.06 |
| Salween | 30.02 |
| Qaidam | 15.59 |
| Hexi Corridor | 6.70 |
| Mekong | 6.62 |
| Ganges | 6.55 |
| Indus | 5.34 |
| Tarim | 0.47 |
| **Total** | **426.59** |

**TABLE S2 | Details for the data sources utilized in this study.**

| **Variable** | **Time span** | **Temporal resolution** | **Spatial resolution** | **Data source** |
| --- | --- | --- | --- | --- |
| ET | 2000.1-2021.8 | Monthly | 500 m | <https://developers.google.cn/earth-engine/datasets/catalog/MODIS_061_MOD16A2GF#terms-of-use> |
| PET | 2000.1-2021.8 | Monthly | 1 km | <https://data.tpdc.ac.cn/zh-hans/data/8b11da09-1a40-4014-bd3d-2b86e6dccad4> |
| SM | 2000.1-2021.8 | Monthly | 1 km | <https://data.tpdc.ac.cn/zh-hans/data/30131436-88d1-4be3-8e3d-14905a29d6d6> |
| Pre | 2000.1-2021.8 | Monthly | 1 km | <https://data.tpdc.ac.cn/zh-hans/data/faae7605-a0f2-4d18-b28f-5cee413766a2> |
| Tmp | 2000.1-2021.8 | Monthly | 1 km | <https://data.tpdc.ac.cn/zh-hans/data/71ab4677-b66c-4fd1-a004-b2a541c4d5bf> |
| LST | 2000.1-2021.8 | Monthly | 1 km | <https://data.tpdc.ac.cn/zh-hans/data/d09ebfe3-a8c9-49b5-aa22-1fd484751d70> |
| GPP | 2000.2-2021.8 | Monthly | 1 km | <https://developers.google.com/earth-engine/datasets/catalog/UMT_NTSG_v2_LANDSAT_GPP?hl=en#description> |
| NDVI | 2000.1-2021.8 | Monthly | 250 m | <https://data.tpdc.ac.cn/zh-hans/data/10535b0b-8502-4465-bc53-78bcf24387b3> |
| NPP | 2000-2021 | Yearly | 250 m & 500 m | <https://data.tpdc.ac.cn/zh-hans/data/37d61044-9802-4d00-96a1-ff331454f265>；<https://lpdaac.usgs.gov/products/mod17a3hgfv061/> |
| NL | 2000-2021 | Yearly | 1 km | <http://www.geodata.cn/data/datadetails.html?dataguid=54416522887785> |
| POP | 2000-2021 | Yearly | 1 km | <https://hub.worldpop.org/geodata/listing?id=74> |

**Abbreviation:** Evapotranspiration (ET); Potential Evapotranspiration (PET); Surface Soil Moisture (SM); Precipitation (Pre); Temperature (Tmp); Land Surface Temperature (LST); Gross Primary Production (GPP); Normalized Difference Vegetation Index (NDVI); Net Primary Production (NPP); Night-time Light (NL); Population Density (POP).
