# Supplementary material for: Groundwater Rise Sustains the World's Largest Alpine Water System Under Global Warming
Source: Adv Sci (Weinh). 2026 Aug 5;13(60):e76957. doi: 10.1002/advs.76957 (PMC13440378; doi:10.1002/advs.76957)
Supplement: Supplementary file 3 [file ADVS-13-e76957-s003.docx]

The supporting text for supporting movie

The movie shows the dynamic changes in the annual mean shallow groundwater depth in the study area from 2000 to 2020. It clearly shows an overall shallowing of groundwater depth across the study area, especially a pronounced shallowing trend in the Inner Plateau. The specific year is indicated at the top of the movie, and the legend ranges from green to red to represent groundwater depth from shallow to deep.
